# COVID-19 Vaccination Coverage among 42,565 Adults Amid the Spread of Omicron Variant in Beijing, China

**DOI:** 10.3390/vaccines11040739

**Published:** 2023-03-27

**Authors:** Chenyuan Qin, Min Du, Yaping Wang, Mingyue Li, Hao Wu, Shugang Li, Jue Liu

**Affiliations:** 1 School of Public Health, Peking University, Xueyuan Road No. 38, Haidian District, Beijing 100191, China; 2 School of General Practice and Continuing Education, Capital Medical University, Xitoutiao Youanmenwai No. 10, Fengtai District, Beijing 100069, China; 3 School of Public Health, Capital Medical University, Xitoutiao Youanmenwai No. 10, Fengtai District, Beijing 100069, China; 4 Key Laboratory of Reproductive Health, National Health and Family Planning Commission of the People’s Republic of China, Peking University, Xueyuan Road No. 38, Haidian District, Beijing 100191, China; 5 Global Center for Infectious Disease and Policy Research & Global Health and Infectious Diseases Group, Peking University, Xueyuan Road No. 38, Haidian District, Beijing 100191, China

**Keywords:** COVID-19, vaccination, sociodemographic determinants, China

## Abstract

Vaccines against coronavirus disease 2019 (COVID-19) have been in use for over two years, but studies that reflect real-world vaccination coverage and demographic determinants are lacking. Using a multistage stratified random cluster sampling method, we planned to directly explore vaccination coverage and the demographic determinants of different doses of COVID-19 vaccines in Beijing, especially in older populations. All 348 community health service centers in 16 districts were involved. We performed multivariable logistic regression analyses to identify demographic determinants of different coverage rates via adjusted odds ratios (aORs) and 95% CIs. Of the 42,565 eligible participants, the total vaccination coverage rates for ≥1 dose, ≥2 doses, ≥3 doses, and 4 doses were 93.3%, 91.6%, 84.9%, and 13.0%, respectively, but decreased to 88.1%, 85.1%, 76.2%, and 3.8% in the older population. Among all participants, younger (aOR = 1.77, 95% CI: 1.60–1.95), male (aOR = 1.15, 95% CI: 1.06–1.23), and better-educated residents (high school and technical secondary school aOR = 1.58, 95% CI: 1.43–1.74; bachelor’s degree aOR = 1.53, 95% CI: 1.37–1.70) were more likely to be fully vaccinated. People who lived in rural areas (aOR = 1.45, 95% CI: 1.31–1.60) and held the new rural cooperative health insurance (aOR = 1.37, 95% CI: 1.20–1.57) established a higher rate of full vaccination coverage. No history of chronic disease was positively associated with a higher coverage rate (aOR = 1.81, 95% CI: 1.66–1.97). Occupation also affected vaccination coverage. Demographic factors influencing the rate of vaccination with at least one or three doses were consistent with the results above. Results remained robust in a sensitivity analysis. Given the highly transmissible variants and declining antibody titers, accelerating the promotion of booster vaccination coverage, especially in high-risk groups such as the elderly, is a top priority. For all vaccine-preventable diseases, rapidly clarifying vaccine-hesitant populations, clearing barriers, and establishing a better immune barrier can effectively safeguard people’s lives and property and coordinate economic development with epidemic prevention and control.

## 1. Introduction

Since the emergence of severe acute respiratory syndrome coronavirus 2 (SARS-CoV-2) in late 2019, coronavirus disease 2019 (COVID-19) has ravaged the world for more than three years [1]. According to the World Health Organization (WHO), 757,264,511 confirmed cases and 6,850,549 deaths have been reported globally as of 28 February 2023 [2]. Five SARS-CoV-2 variants (Alpha, Beta, Gamma, Delta, and Omicron) have been added to the list of variants of concern (VOCs) to suggest their great threat to human health and life [3]. It has been well documented that confirmed cases and deaths continue to rise markedly with the emergence of the Omicron (B1.1.519) variant and its subvariants [4,5]. After experiencing a massive epidemic in February 2020, comprehensive measures have been made to minimize the health, economic, and social impacts since May 2020 [6]. Due to the universalization of COVID-19 vaccination, accumulation of experience in prevention and control, and the virus’s mutation toward lower pathogenicity and possible long-term existence in nature, China has entered a new stage, and the “*Twenty optimized measures*”, “*Ten new measures*”, and “*Class-B management of COVID-19*” have been announced successively in China [7,8]. With the slowdown of the policies, Omicron sublineages BA.5.2 and BF.7 with high infectivity and low toxicity caused a new round of epidemic in China at the end of 2022 [9].

Vaccination is considered to be the most effective and economical strategy to prevent and control the epidemic of infectious diseases [10,11,12,13,14]. Studies have demonstrated the significant role of vaccines in reducing the risk of symptomatic infections, severe illness, and death [15,16]. Governments have formulated and continuously adjusted vaccination strategies in a timely and scientific manner in accordance with the situation of the epidemic and the progress of vaccine research and development [17,18,19,20,21,22,23,24,25,26]. Although vaccine hesitancy is a common phenomenon globally, previous studies have shown that the acceptance of COVID-19 vaccines is generally high in Asia, especially in China [27,28]. The two-dose inactivated vaccines (Sinovac-CoronaVac COVID-19 vaccine, and Sinopharm BBIBP vaccine) are the most widely used COVID-19 vaccines in China [29]. Since December 2020, the primary series of vaccination programs have been launched in a sequential manner in key populations at high risk of infection, including people aged 18–59 years, people aged 60 years and above, and people aged 3–17 years [17]. In October 2021, the first dose booster vaccination and sequential booster vaccination, and second dose booster vaccination were launched in December 2022 [17,30]. Vaccine doses are significantly associated with severe outcomes [15,31]. The relative risk of dying from COVID-19 was 33.2 times higher in unvaccinated individuals than in those who received ≥2 doses of COVID-19 vaccines [31]. Among the elderly (≥60 years), the relative risk of death was 21.3 times and 2.3 times higher in unvaccinated individuals than in those who received ≥2 doses or received 1 dose, respectively [31]. With the downgrading of COVID-19 management and a shift from preventing infections to preventing severe diseases in China, the number of infection cases increased, which brought a certain challenge to medical and health service resources [32]. It is particularly important to clarify the coverage of different doses of COVID-19 vaccines and their distribution in populations with different demographic characteristics for better remediation.

To our knowledge, the vast majority of previous studies on vaccine acceptability have focused on vaccination intentions and influencing factors, including age, gender, income level, marital status, education level, rural environment, occupation, health insurance, etc. [33,34,35,36,37,38,39,40,41,42] Studies that reflect real-world vaccination coverage and demographic determinants are lacking. Therefore, we conducted a quick survey during the latest outbreak based on all community health service centers (CHSCs) in the capital of China to fill the current research gap. Our study may contribute to clarifying the current status of COVID-19 vaccination in China, exploring the potential for future outbreaks and the possible disease, resource, and economic burden, and specifying the direction of rollout and implementation of the next dose of COVID-19 vaccines or future vaccination programs for other pandemics in priority populations. The demographic characteristics that affect the actual vaccine coverage rate are universal and can be used as a reference for other similar vaccine-preventable diseases and countries with similar national conditions to promote vaccination from the simplest and most direct perspective. Additionally, our results may also provide basic data support for modeling studies based on the current status of real-world vaccination or correlation with disease outcomes after SARS-CoV-2 infection.

## 2. Materials and Methods

### 2.1. Study Design, Participants, and Data Collection

This cross-sectional study was conducted from 26 December to 31 December 2022, in collaboration with the Beijing Municipal Health Commission and 16 district-level health commissions. The inclusion criteria were as follows: (1) ≥18 years old; (2) living in Beijing for more than 6 months; and (3) at least one family member signed a family doctor service contract with CHSCS. PASS software 15.0 (NCSS LLC., Kaysville, UT, USA) was used to calculate the minimum sample size with a coverage rate ranging from 10% to 100%, an α of 0.05, and a two-sided confidence interval width of 0.02, using the exact (Clopper–Pearson) method. The minimum sample size was 9701. Considering the nature of cluster sampling, we expanded the sample size.

We used a multistage stratified random cluster sampling method to recruit a representative sample of approximately 43,000 individuals. In the first stage, we selected all 16 districts in Beijing, and the number of recruits was proportional to the number of permanent residents in each district. In the second stage, the target survey population of each CHSC was assigned based on their service circle. All 348 CHSCs in 16 districts were involved. In the third stage, households were randomly selected from the CHSC family doctor contract system. All members in the selected households who met the inclusion criteria were consecutively enrolled. Electronic questionnaires were distributed through WeChat groups established in advance at each community health service center. For those who could not respond independently via the online survey (e.g., older adults without smartphones), phone interviews were conducted by trained community health workers. Respondents who submitted questionnaires agreed to participate in the study. Finally, 42,565 eligible respondents were included in the analysis, yielding an overall response rate of 99.0%.

This study met the requirements of the Declaration of Helsinki and was approved by the Ethics Committee of Peking University (IRB00001052-21126).

### 2.2. Questionnaire Design

Gender, age, location, education level, health insurance, occupation, and self-reported chronic disease history (whether or not diagnosed by a physician as having hypertension, diabetes, dyslipidemia, heart disease, stroke, and cerebrovascular disease, bronchitis, emphysema, asthma or pneumonia, tuberculosis, gastrointestinal disease, immunodeficiency disease, arthritis/rheumatic or rheumatoid, chronic kidney disease, hepatitis or cancer) were all investigated.

We set separate questions to ask about COVID-19 vaccination status, including “unvaccinated”, “1 dose”, “2 doses”, “3 doses”, and “4 doses”. The inactivated vaccines (Sinovac-CoronaVac COVID-19 vaccine, and Sinopharm BBIBP vaccine) are the most widely used COVID-19 vaccines in China, covering almost all recipients [29].

### 2.3. Definition of Four COVID-19 Vaccination Coverage Rates

The COVID-19 vaccination coverage rate was the primary outcome of this study. Based on the results of the questionnaire on vaccination status, we divided COVID-19 vaccination coverage into four categories: ≥1 dose; ≥2 doses; ≥3 doses, and 4 doses. Taking the coverage rate of ≥1 dose of COVID-19 vaccines as an example, the numerator was the number of residents who received at least 1 dose, and the denominator was the number of residents who did not. The other three categories of vaccination coverage rates were calculated with similar formulas separately. Notably, the coverage rate of 4 doses only refers to the proportion of people who have received a total of four doses of COVID-19 vaccines in the whole population. Completion of ≥2 doses of COVID-19 vaccines was considered to finish the primary vaccination series (full vaccination), and completion of ≥3 doses was considered to complete the first booster immunization. The fourth dose usually means a second booster dose.

### 2.4. Statistical Analysis

Frequencies and percentages were used to describe the basic characteristics of the study population. We calculated the COVID-19 vaccination coverage rates (≥1 dose; ≥2 doses; ≥3 doses, and 4 doses) and 95% confidence intervals (CIs) for the overall population, for persons 18 to 59 years of age, and for persons 60 years of age or older. Independent chi-square tests and Cochran–Armitage tests for trends were used to compare the vaccination rates among groups stratified by demographic characteristics. We performed multivariable logistic regression analyses to identify demographic determinants of different coverage rates. Adjusted odds ratios (aORs) and 95% CIs were both calculated. The Hosmer and Lemeshow test was used to assess the goodness of model fitting. Age was considered a continuous variable in sensitivity analyses to verify the robustness of the results. All statistical analyses were performed using SPSS 26.0 (IBM SPSS Inc., New York, NY, USA). Two-sided *p* ≤ 0.05 were considered to be statistically significant.

## 3. Results

### 3.1. Basic Characteristics of the Study Population

Table 1 presents the basic demographic characteristics and vaccination status of the study population. Of the 42,565 eligible participants, 65.8% (27,990) were female, 70.9% (30,175) lived in urban areas, and 55.0% (23,419) had at least a bachelor’s degree. Nearly 80% of the participants were covered by medical insurance for urban employees and residents, and the proportions of healthcare workers and retirees were both over 20% in this survey.

Among all participants, 12,530 (29.4%) people were aged 60 years and above. For the elderly population (≥60 years), 37.0% (4638) were at least 70 years old, and the difference between the proportions of males and females was significantly smaller than that of the whole population (17.2% vs. 31.6%). The overall education level of the elderly is relatively lower, and nearly half of them have only a junior high school education or below. Interestingly, the number of people with chronic diseases was almost the same as those without chronic diseases in all participants, but the former accounted for 83.8% (10,500/12,530) of the elderly population.

### 3.2. COVID-19 Vaccination Coverage Rates

Four types of vaccination coverage rates are shown in Table 2. For all populations, the total vaccination coverage rates were 93.3% (95% CI: 93.1–93.6%) for at least one dose, 91.6% (93.1–93.6%) for at least two doses, 84.9% (93.1–93.6%) for at least three doses, and 13.0% (93.1–93.6%) for 4 doses. For the elderly population, the total vaccination coverage rates for ≥ 1 dose, ≥ 2 doses, ≥ 3 doses, and 4 doses decreased to 88.1% (87.6–88.7%), 85.1% (84.5–85.7%), 76.2% (75.4–76.9%), and 3.8% (3.5–4.2%), respectively. For people aged 18–59 years old, the four abovementioned rates were higher, as shown in Table S1.

Table 2 and Table S1 also present the four coverage rates among the three age groups stratified by demographic characteristics. As shown in Table 2, in group 1, people who were younger, in rural areas, and well-educated generally had higher vaccine coverage rates. Healthcare workers had the highest coverage rates across all occupational categories. The coverage rates of civil servants and employees of enterprises/institutions who received the fourth dose were higher than those of service industry workers, which was contrary to the results of the other three vaccination coverage rates. Having a history of chronic disease generally implied relatively lower vaccine coverage.

In the elderly population, the vaccine coverage rate was higher among participants with new rural cooperative medical insurance, which differed from the results in group 1. For the fourth dose (also known as the second booster dose), the coverage rate increased with education (P_trend_ < 0.05) and was higher among men than among women (Table 2).

### 3.3. Demographic Determinants Associated with Vaccination Coverage Rates

Table 3 shows the demographic determinants associated with the COVID-19 vaccination coverage rates among the entire population and people aged ≥60 years old.

In group 1, younger (aOR = 1.77, 95% CI: 1.60–1.95), male (aOR = 1.15, 95% CI: 1.06–1.23) and better-educated residents (high school and technical secondary school aOR = 1.58, 95% CI: 1.43–1.74; bachelor’s degree aOR = 1.53, 95% CI: 1.37–1.70) were more likely to be fully vaccinated. People who lived in rural areas (aOR = 1.45, 95% CI: 1.31–1.60) and held the new rural cooperative health insurance (aOR = 1.37, 95% CI: 1.20–1.57) established a higher rate of full vaccination coverage than those who lived in urban areas. No history of chronic disease was positively associated with a higher coverage rate (aOR = 1.81, 95% CI: 1.66–1.97). Civil servants and employees of enterprises/institutions, pleasant, retirees, and freelance workers had lower vaccination coverage (≥2 doses) than those in the service industry, whereas the reverse was true for healthcare workers. The demographic factors influencing the rate of vaccination with at least one or three doses were consistent with the results above. In addition, all demographic characteristics included in the model, except gender and chronic disease history, affected the fourth injection rate of residents. It is worth noting that civil servants and employees of enterprises/institutions (aOR = 1.30, 95% CI: 1.13–1.50) showed a higher four-dose vaccination rate than service industry personnel. The vaccination rate of residents protected by the new rural cooperative medical insurance was lower than that of residents protected by urban medical insurance (aOR = 0.78, 95% CI: 0.67–0.90).

For the vaccination rates (≥1 dose; ≥2 doses; ≥3 doses and 4 doses) in the older population, lower age, living in rural areas, better level of education, and healthcare workers were all significant positive demographic determinants. Compared with participants with chronic disease, those without chronic disease who received at least one dose (aOR = 1.85, 95% CI: 1.54–2.23), at least two doses (aOR = 1.51, 95% CI: 1.29–1.77), and at least three doses (aOR = 1.51, 95% CI: 1.33–1.71) increased significantly, but this result did not apply to the coverage of the fourth injection. The type of health insurance was not associated with the four-dose coverage but with the coverage of the other three vaccination groups. The vaccination coverage rate of the unemployed/freelance was lower than that of service workers (≥2 doses aOR = 0.60, 95% CI: 0.41–0.87; ≥3 doses aOR = 0.68, 95% CI: 0.49–0.93; 4 doses aOR = 0.27, 95% CI: 0.14–0.55). Men were 1.38 (95% CI: 1.15–1.67) times more likely to receive the fourth dose than women.

Demographic determinants associated with COVID-19 vaccination coverage among people aged 18–59 years old are presented in Table S2. The model results remained robust by bringing age as a continuous variable in the sensitivity analysis (Table S3).

## 4. Discussion

To our knowledge, few studies have directly reported vaccination coverage and the demographic determinants of different doses of COVID-19 vaccines, especially in the older population. According to our results, 91.6% of the 42,565 eligible participants were fully vaccinated (≥2 doses), and 84.9% were at least administered the first booster dose (≥3 doses). For the 12,530 older people, the coverage rates of full vaccination and the first booster dose decreased to 85.1% and 76.2%, respectively. The second booster vaccination is in the early stages of being rolled out. Age, location, education, health insurance, occupation, and chronic disease history were all demographic determinants of vaccine coverage rates. Gender played a more significant role in all populations than in the elderly population, and older men were more likely to receive a second booster dose. Our study reveals the real-world vaccination coverage and demographic distribution of COVID-19 vaccines in Beijing two years after vaccine introduction, which may clarify the publicity and implementation direction for the next dose of COVID-19 vaccines or future vaccination programs for other pandemics in priority populations. The demographic characteristics that affect the actual vaccine coverage rate are universal and can be used as a reference for other similar vaccine-preventable diseases and countries with similar national conditions to promote vaccination from the simplest and most direct perspective. Additionally, our results may also provide basic data support for modeling studies based on the current status of real-world vaccination or correlation with disease outcomes after SARS-CoV-2 infection.

Vaccination is considered to be the most effective and economical strategy to prevent and control the epidemic of infectious diseases [10,11,12,13,14]. In our study, we found that 93.3% of the population was vaccinated with at least one dose, 91.6% were fully vaccinated, and 84.9% were administered at least the first booster dose. The booster dose coverage rate was lower than the 91.61% of people who were willing to be vaccinated in a previous large national survey [43]. According to official data from the Centers for Disease Control and Prevention (CDC) in China, as of 30 January 2023, the coverage rates of ≥ 1 dose and ≥ 2 doses reached 92.9% and 90.6%, respectively, which was basically consistent with our research [44]. However, the coverage rate of the first booster dose that we investigated was much higher than the 60.2% reported by the Joint Prevention and Control Mechanism of the State Council on 23 February 2023 [17]. This may be attributed to the strong capacity of vaccine production and stockpiling, sufficient publicity and mobilization, and the efforts of politicians, medical personnel, community health workers, and residents. For the elderly population, the coverage rates of the first dose, full vaccination, and the first booster dose of COVID-19 vaccines in our study decreased to 88.1%, 85.1%, and 76.2%, respectively. Studies have shown that 82.8% of elderly individuals in China were willing to receive COVID-19 vaccine booster injections, which was much higher than that in Jordan and Bangladesh [45,46,47]. Based on the national elderly population survey by the end of 2022, 96.6% were covered by the full vaccination (≥2 doses), and 92.2% were covered by the first booster dose [44]. Vaccine coverage rates of the older population in this study were slightly below the national level, which may be related to the different definitions [44]. For the primary series, an earlier global meta-analysis of 9753 older adults showed that the prevalence of unwillingness to vaccinate against COVID-19 was 27.03%, whilst the uncertainty was 19.33% [48]. In another national study, the vaccination willingness of the elderly over 60 years old was 87.68%, which was basically the same as the actual vaccination rate in this study, but significantly higher than the global average [43,48]. A total of 82.8% of Chinese elderly were willing to receive the first booster dose, which was slightly higher than the vaccination coverage in this study [49]. The booster doses of vaccination promotion need to be strengthened in both the general population and the elderly population. Although China has thus far built up a relatively solid immune barrier through vaccination and natural infection, the authorities should make scientific plans for possible future vaccination strategies, considering the decline of antibody titers over time and the continuous evolution of the virus [50,51,52].

According to our results, age was negatively associated with COVID-19 vaccine coverage in both the whole population and the elderly population. It is well known that old age is a high-risk factor for poor prognosis of various diseases, including severe illness and death after SARS-CoV-2 infection [53,54]. Another key issue to note is that older people generally produce lower levels of effective antibodies after vaccination than younger people [54,55]. The older population is the priority group for COVID-19 vaccination, and improving and ensuring adequate antibody titers is a priority [49,56]. Advanced age and multiple comorbidities are risk factors for the development of long COVID-19, and effective vaccination may reduce the chances of long-term sequelae, as a cohort study suggested [45,57]. Chronic disease history can also reduce vaccine coverage [42]. Apart from vaccine contraindications confirmed by doctors, there may also be excessive concerns about the safety of vaccines and misjudgments about one’s own health status [58]. Residents in rural areas and with new rural cooperative health insurance were more likely to receive COVID-19 vaccines in this study, which may be because of their greater tendency to believe in official propaganda and compliance with government arrangements [59]. However, previous studies have demonstrated lower vaccine acceptance in rural settings [42]. Healthcare workers were more supportive of COVID-19 vaccines than non-healthcare workers, which is consistent with our results [40,42,60]. Vaccine coverage among unemployed/freelancers is of concern, especially in the context of rising unemployment during the pandemic [61]. The effect of gender differences on vaccination coverage was significant in the whole population, and men were more likely to have high vaccine coverage. Similar results were obtained in a systematic review exploring the impact on vaccination intentions [42]. However, this association was not significant in the elderly population. A pandemic similar to COVID-19 could occur at any time, posing a serious threat to the security and public health of all countries [62]. For vaccine-preventable diseases, good governance can support countries in preparing for social and public measures such as effective and democratic vaccination campaigns, which contribute to maintaining social stability and ensuring smooth economic development [12,63,64].

The limitations of this study are as follows. First, although this study adopted multistage stratified cluster sampling to cover all the CHSCs in all 16 administrative districts of Beijing, China, bias is hard to avoid. Therefore, we chose to expand the sample size as much as our financial and human resources allowed. More than 42,000 people were eventually enrolled. Second, because the proportion of females in our included population was significantly higher than that of males, we may have underestimated the vaccine coverage in the total population to some extent based on the analysis of the effect of demographic characteristics on vaccination rates in this study. In addition, caution is needed when extrapolating the results. Third, the vaccination status and demographic characteristics were obtained through self-report of the respondents, which may cause recall bias. Fourth, given that information about infection status was not available at that time, we have not yet further explored the association between vaccination status and COVID-19 outcomes in such a large population. Nevertheless, this is the first real-world study of a large sample in a contracted population of family physicians in a community health service center in China. The results filled the research gap, that is, few large sample studies have published the status of residents’ vaccination and its determinants of demographic characteristics in such detail. Because this is a quick survey with a large sample size, more demographic characteristics, such as income, marriage, and childbirth, were not examined, which further research should take seriously. In addition, studies on the association between vaccination and infection outcomes based on real-world data are also feasible research directions.

## 5. Conclusions

In conclusion, the population coverage rate of the primary vaccination series has reached a high level in China, while the booster coverage still needs to be improved, especially in the older population. Age, location, education, health insurance, occupation, and chronic disease history were all demographic determinants of vaccine coverage. Gender played a more significant role in all populations than in the elderly population. It is more targeted and practical to carry out vaccination promotion based on demographic characteristics according to local conditions. For all vaccine-preventable diseases, rapidly clarifying vaccine-hesitant populations, clearing barriers, and establishing a better immune barrier can effectively safeguard people’s lives and property and coordinate economic development with epidemic prevention and control.

## Figures and Tables

**Table 1:** Basic characteristics of the included study population.

Characteristics	Total (N = 42,565)		18–59 Years (N = 30,035)		≥60 years (N = 12,530)	
	n	Percentage (%)	n	Percentage (%)	n	Percentage (%)
**Age (years)**						
18–59	30,035	70.6	-	-	-	-
≥60	12,530	29.4	-	-	-	-
18–29	-	-	4158	13.8	-	-
30–39	-	-	9514	31.7	-	-
40–49	-	-	8226	27.4	-	-
50–59	-	-	8137	27.1	-	-
60–64	-	-	-	-	3007	24.0
65–69	-	-	-	-	4885	39.0
≥70	-	-	-	-	4638	37.0
**Gender**						
Male	14,575	34.2	9386	31.3	5189	41.4
Female	27,990	65.8	20,649	68.7	7341	58.6
**Location**						
Urban	30,175	70.9	21,609	71.9	8566	68.4
Rural	12,390	29.1	8426	28.1	3964	31.6
**Education**						
Junior high school and below	9728	22.9	3935	13.1	5793	46.2
High school and technical secondary school	9418	22.1	5177	17.2	4241	33.8
Bachelor’s degree	21,149	49.7	18,786	62.5	2363	18.9
Master’s degree or above	2270	5.3	2137	7.1	133	1.1
**Health insurance**						
Medical insurance for urban employees and residents	33,967	79.8	25,514	84.9	8453	67.5
New rural cooperative medical insurance	6031	14.2	2864	9.5	3167	25.3
Others	2567	6.0	1657	5.5	910	7.3
**Occupation**						
Service industry personnel	3572	8.4	3194	10.6	378	3
Healthcare workers	10,824	25.4	10,321	34.4	503	4
Civil servants and employees of enterprises/institutions	5680	13.3	5322	17.7	358	2.9
Pleasant	3995	9.4	1831	6.1	2164	17.3
Retired	9472	22.3	1860	6.2	7612	60.8
Unemployed/freelance	2460	5.8	1859	6.2	601	4.8
Others	6562	15.4	5648	18.8	914	7.3
**Chronic disease**						
No	21,559	50.6	19,529	65	2030	16.2
Yes	21,006	49.4	10,506	35	10,500	83.8

**Table 2:** COVID-19 vaccination coverage rates stratified by dose among all populations and people aged ≥60 years old.

Characteristics	≥1 Dose			≥2 Doses			≥3 Doses			4 Doses		
	n (%)	95% CI	*p* Value	n (%)	95% CI	*p* Value	n (%)	95% CI	*p* Value	n (%)	95% CI	*p* Value
**Group 1: All populations**												
**Total**	39,731 (93.3)	93.1–93.6		38,996 (91.6)	91.4–91.9		36,119 (84.9)	84.5–85.2		5530 (13.0)	12.7–13.3	
**Age (years)**			**<0.001**			**<0.001**			**<0.001**			**<0.001**
18–59	28,686 (95.5)	95.3–95.7		28,333 (94.3)	94.1–94.6		26,574 (88.5)	88.1–88.8		5050 (16.8)	16.4–17.2	
≥60	11,045 (88.1)	87.6–88.7		10,663 (85.1)	84.5–85.7		9545 (76.2)	75.4–76.9		480 (3.8)	3.5–4.2	
**Gender**			0.523			0.225			0.716			**<0.001**
Male	13,589 (93.2)	92.8–93.6		13,320 (91.4)	90.9–91.8		12,355 (84.8)	84.2–85.3		1651 (11.3)	10.8–11.8	
Female	26,142 (93.4)	93.1–93.7		25,676 (91.7)	91.4–92.1		23,764 (84.9)	84.5–85.3		3879 (13.9)	13.5–14.3	
**Location**			**<0.001**			**<0.001**			**<0.001**			0.499
Urban	28,033 (92.9)	92.6–93.2		27,494 (91.1)	90.8–91.4		25,366 (84.1)	83.6–84.5		3899 (12.9)	12.5–13.3	
Rural	11,698 (94.4)	94.0–94.8		11,502 (92.8)	92.4–93.3		10,753 (86.8)	86.2–87.4		1631 (13.2)	12.6–13.8	
**Education**			**<0.001**			**<0.001**			**<0.001**			**<0.001**
Junior high school and below	8702 (89.5)	88.8–90.1		8404 (86.4)	85.7–87.1		7626 (78.4)	77.6–79.2		453 (4.7)	4.3–5.1	
High school and technical secondary school	8718 (92.6)	92.0–93.1		8577 (91.1)	90.5–91.6		7959 (84.5)	83.8–85.2		770 (8.2)	7.6–8.7	
Bachelor’s degree	20,168 (95.4)	95.1–95.6		19,909 (94.1)	93.8–94.4		18,644 (88.2)	87.7–88.6		4042 (19.1)	18.6–19.6	
Master’s degree or above	2143 (94.4)	93.4–95.3		2106 (92.8)	91.7–93.8		1890 (83.3)	81.7–84.8		265 (11.7)	10.4–13.0	
**Health insurance**			**0.001**			**0.001**			**<0.001**			**<0.001**
Medical insurance for urban employees and residents	31,762 (93.5)	93.2–93.8		31,195 (91.8)	91.5–92.1		29,002 (85.4)	85.0–85.8		4958 (14.6)	14.2–15.0	
New rural cooperative medical insurance	5615 (93.1)	92.4–93.7		5493 (91.1)	90.3–91.8		5065 (84.0)	83.0–84.9		368 (6.1)	5.5–6.7	
Others	2354 (91.7)	90.6–92.7		2308 (89.9)	88.7–91.0		2052 (79.9)	78.4–81.5		204 (7.9)	6.9–9.0	
**Occupation**			**<0.001**			**<0.001**			**<0.001**			**<0.001**
Service industry personnel	3417 (95.7)	95.0–96.3		3381 (94.7)	93.9–95.4		3161 (88.5)	87.4–89.5		367 (10.3)	9.3–11.3	
Healthcare workers	10,551 (97.5)	97.2–97.8		10,431 (96.4)	96.0–96.7		9999 (92.4)	91.9–92.9		3090 (28.5)	27.7–29.4	
Civil servants and employees of enterprises/institutions	5388 (94.9)	94.3–95.4		5319 (93.6)	93.0–94.3		4952 (87.2)	86.3–88.0		760 (13.4)	12.5–14.3	
Pleasant	3726 (93.3)	92.5–94.0		3636 (91.0)	90.1–91.9		3374 (84.5)	83.3–85.6		227 (5.7)	5.0–6.4	
Retired	8306 (87.7)	87.0–88.3		8088 (85.4)	84.7–86.1		7258 (76.6)	75.8–77.5		301 (3.2)	2.8–3.5	
Unemployed/freelance	2181 (88.7)	87.4–89.9		2121 (86.2)	84.8–87.5		1882 (76.5)	74.8–78.1		93 (3.8)	3.1–4.6	
Others	6162 (93.9)	93.3–94.5		6020 (91.7)	91.1–92.4		5493 (83.7)	82.8–84.6		692 (10.5)	9.8–11.3	
**Chronic disease**			**<0.001**			**<0.001**			**<0.001**			**<0.001**
No	20,789 (96.4)	96.2–96.7		20,512 (95.1)	94.9–95.4		19,159 (88.9)	88.4–89.3		3576 (16.6)	16.1–17.1	
Yes	18,942 (90.2)	89.8–90.6		18,484 (88.0)	87.5–88.4		16,960 (80.7)	80.2–81.3		1954 (9.3)	8.9–9.7	
**Group 2: ≥ 60 years old**												
**Total**	11,045 (88.1)	87.6–88.7		10,663 (85.1)	84.5–85.7		9545 (76.2)	75.4–76.9		480 (3.8)	3.5–4.2	
**Age (years)**			**<0.001**			**<0.001**			**<0.001**			**<0.001**
60–64	2762 (91.9)	90.8–92.8		2711 (90.2)	89.1–91.2		2524 (83.9)	82.6–85.2		146 (4.9)	4.1–5.7	
65–69	4439 (90.9)	90.0–91.7		4316 (88.4)	87.4–89.2		3884 (79.5)	78.4–80.6		224 (4.6)	4.0–5.2	
≥70	3844 (82.9)	81.8–83.9		3636 (78.4)	77.2–79.6		3137 (67.6)	66.3–69.0		110 (2.4)	2.0–2.8	
**Gender**			0.116			0.44			0.368			**<0.001**
Male	4602 (88.7)	87.8–89.5		4431 (85.4)	84.4–86.3		3974 (76.6)	75.4–77.7		236 (4.5)	4.0–5.1	
Female	6443 (87.8)	87.0–88.5		6232 (84.9)	84.1–85.7		5571 (75.9)	74.9–76.9		244 (3.3)	2.9–3.8	
**Location**			**<0.001**			**<0.001**			**<0.001**			**<0.001**
Urban	7421 (86.6)	85.9–87.3		7160 (83.6)	82.8–84.4		6408 (74.8)	73.9–75.7		277 (3.2)	2.9–3.6	
Rural	3624 (91.4)	90.5–92.3		3503 (88.4)	87.3–89.3		3137 (79.1)	77.9–80.4		203 (5.1)	4.5–5.8	
**Education**			**<0.001**			**<0.001**			**<0.001**			**<0.001**
Junior high school and below	5008 (86.4)	85.5–87.3		4754 (82.1)	81.1–83.0		4168 (71.9)	70.8–73.1		157 (2.7)	2.3–3.2	
High school and technical secondary school	3822 (90.1)	89.2–91.0		3744 (88.3)	87.3–89.2		3403 (80.2)	79.0–81.4		182 (4.3)	3.7–4.9	
Bachelor’s degree	2096 (88.7)	87.4–89.9		2050 (86.8)	85.3–88.1		1872 (79.2)	77.6–80.8		128 (5.4)	4.6–6.4	
Master’s degree or above	119 (89.5)	83.4–93.8		115 (86.5)	79.9–91.5		102 (76.7)	69.0–83.3		13 (9.8)	5.6–15.7	
**Health insurance**			**<0.001**			**<0.001**			**<0.001**			**0.003**
Medical insurance for urban employees and residents	7355 (87.0)	86.3–87.7		7088 (83.9)	83.1–84.6		6333 (74.9)	74.0–75.8		289 (3.4)	3.0–3.8	
New rural cooperative medical insurance	2898 (91.5)	90.5–92.4		2805 (88.6)	87.4–89.6		2519 (79.5)	78.1–80.9		149 (4.7)	4.0–5.5	
Others	792 (87.0)	84.7–89.1		770 (84.6)	82.2–86.8		693 (76.2)	73.3–78.8		42 (4.6)	3.4–6.1	
**Occupation**			**<0.001**			**<0.001**			**<0.001**			**<0.001**
Service industry personnel	335 (88.6)	85.1–91.5		331 (87.6)	84.0–90.6		301 (79.6)	75.4–83.5		30 (7.9)	5.5–11.0	
Healthcare workers	482 (95.8)	93.8–97.3		474 (94.2)	91.9–96.0		453 (90.1)	87.2–92.4		88 (17.5)	14.4–21.0	
Civil servants and employees of enterprises/institutions	318 (88.8)	85.3–91.8		312 (87.2)	83.4–90.3		290 (81.0)	76.7–84.8		21 (5.9)	3.8–8.7	
Pleasant	1990 (92.0)	90.8–93.0		1919 (88.7)	87.3–90.0		1729 (79.9)	78.2–81.5		81 (3.7)	3.0–4.6	
Retired	6623 (87.0)	86.2–87.7		6431 (84.5)	83.7–85.3		5729 (75.3)	74.3–76.2		219 (2.9)	2.5–3.3	
Unemployed/freelance	514 (85.5)	82.5–88.2		479 (79.7)	76.3–82.8		420 (69.9)	66.1–73.4		12 (2.0)	1.1–3.4	
Others	783 (85.7)	83.3–87.8		717 (78.4)	75.7–81.0		623 (68.2)	65.1–71.1		29 (3.2)	2.2–4.5	
**Chronic disease**			**<0.001**			**<0.001**			**<0.001**			**0.001**
No	1895 (93.3)	92.2–94.4		1825 (89.9)	88.5–91.2		1689 (83.2)	81.5–84.8		105 (5.2)	4.3–6.2	
Yes	9150 (87.1)	86.5–87.8		8838 (84.2)	83.5–84.9		7856 (74.8)	74.0–75.6		375 (3.6)	3.2–3.9	

Statistically significant *p*-values were bold. Notably, the coverage rate of 4 doses only refers to the proportion of people who have received a total of four doses of COVID-19 vaccines in the whole population. CI: confidence interval.

**Table 3:** Demographic determinants associated with the COVID-19 vaccination coverage rates among all populations and people aged ≥60 years old.

Characteristics	≥1 Dose		≥2 Doses		≥3 Doses		4 Doses	
	aOR (95% CI)	*p* Value	aOR (95% CI)	*p* Value	aOR (95% CI)	*p* Value	aOR (95% CI)	*p* Value
**Group 1: All population**								
**Age (years)**								
18–59	1.54 (1.38–1.72)	**<0.001**	1.77 (1.60–1.95)	**<0.001**	1.73 (1.60–1.88)	**<0.001**	1.83 (1.62–2.08)	**<0.001**
≥60	Reference		Reference		Reference		Reference	
**Gender**								
Male	1.17 (1.08–1.27)	**<0.001**	1.15 (1.06–1.23)	**<0.001**	1.16 (1.09–1.23)	**<0.001**	1.04 (0.97–1.11)	0.281
Female	Reference		Reference		Reference		Reference	
**Location**								
Urban	Reference		Reference		Reference		Reference	
Rural	1.41 (1.27–1.58)	**<0.001**	1.45 (1.31–1.60)	**<0.001**	1.38 (1.28–1.49)	**<0.001**	1.57 (1.46–1.69)	**<0.001**
**Education**								
Junior high school and below	Reference		Reference		Reference		Reference	
High school and technical secondary school	1.45 (1.30–1.61)	**<0.001**	1.58 (1.43–1.74)	**<0.001**	1.47 (1.36–1.59)	**<0.001**	1.40 (1.23–1.60)	**<0.001**
Bachelor’s degree	1.39 (1.24–1.57)	**<0.001**	1.53 (1.37–1.70)	**<0.001**	1.28 (1.18–1.39)	**<0.001**	1.68 (1.47–1.92)	**<0.001**
Master’s degree or above	1.09 (0.87–1.35)	0.465	1.16 (0.95–1.41)	0.138	0.84 (0.73–0.97)	**0.019**	1.04 (0.86–1.25)	0.71
**Health insurance**								
Medical insurance for urban employees and residents	Reference		Reference		Reference		Reference	
New rural cooperative medical insurance	1.31 (1.13–1.53)	**<0.001**	1.37 (1.20–1.57)	**<0.001**	1.21 (1.09–1.35)	**<0.001**	0.78 (0.67–0.90)	**0.001**
Others	0.93 (0.80–1.08)	0.33	0.96 (0.84–1.11)	0.59	0.85 (0.77–0.95)	**0.003**	0.75 (0.65–0.88)	**<0.001**
**Occupation**								
Service industry personnel	Reference		Reference		Reference		Reference	
Healthcare workers	1.63 (1.33–2.02)	**<0.001**	1.36 (1.13–1.64)	**0.001**	1.61 (1.41–1.84)	**<0.001**	3.13 (2.76–3.54)	**<0.001**
Civil servants and employees of enterprises/institutions	0.83 (0.67–1.02)	0.079	0.81 (0.67–0.98)	**0.03**	0.97 (0.85–1.11)	0.69	1.30 (1.13–1.50)	**<0.001**
Pleasant	0.77 (0.61–0.96)	**0.023**	0.73 (0.59–0.89)	**0.002**	0.85 (0.73–1.00)	**0.043**	0.76 (0.62–0.92)	**0.006**
Retired	0.64 (0.53–0.77)	**<0.001**	0.70 (0.59–0.83)	**<0.001**	0.79 (0.69–0.89)	**<0.001**	0.49 (0.41–0.58)	**<0.001**
Unemployed/freelance	0.39 (0.32–0.49)	**<0.001**	0.40 (0.33–0.48)	**<0.001**	0.47 (0.41–0.54)	**<0.001**	0.39 (0.31–0.50)	**<0.001**
Others	0.69 (0.57–0.84)	**<0.001**	0.62 (0.53–0.74)	**<0.001**	0.69 (0.61–0.78)	**<0.001**	1.00 (0.87–1.14)	0.963
**Chronic disease**								
No	2.02 (1.83–2.23)	**<0.001**	1.81 (1.66–1.97)	**<0.001**	1.37 (1.28–1.46)	**<0.001**	1.01 (0.94–1.08)	0.866
Yes	Reference		Reference		Reference		Reference	
**Group 2: ≥60 years old**								
**Age (years)**								
60–64	1.99 (1.70–2.33)	**<0.001**	2.14 (1.85–2.47)	**<0.001**	2.17 (1.92–2.44)	**<0.001**	1.81 (1.39–2.37)	**<0.001**
65–69	1.92 (1.70–2.18)	**<0.001**	1.96 (1.74–2.19)	**<0.001**	1.74 (1.58–1.91)	**<0.001**	1.75 (1.38–2.22)	**<0.001**
≥70	Reference		Reference		Reference		Reference	
**Gender**								
Male	1.12 (1.00–1.26)	**0.0497**	1.06 (0.96–1.18)	0.259	1.07 (0.98–1.17)	0.131	1.38 (1.15–1.67)	**0.001**
Female	Reference		Reference		Reference		Reference	
**Location**								
Urban	Reference		Reference		Reference		Reference	
Rural	1.48 (1.25–1.75)	**<0.001**	1.49 (1.28–1.73)	**<0.001**	1.25 (1.11–1.42)	**<0.001**	1.81 (1.39–2.37)	**<0.001**
**Education**								
Junior high school and below	Reference		Reference		Reference		Reference	
High school and technical secondary school	1.43 (1.24–1.64)	**<0.001**	1.56 (1.37–1.77)	**<0.001**	1.46 (1.31–1.62)	**<0.001**	1.48 (1.16–1.89)	**0.002**
Bachelor’s degree	1.39 (1.17–1.64)	**<0.001**	1.53 (1.31–1.78)	**<0.001**	1.50 (1.32–1.71)	**<0.001**	2.15 (1.60–2.88)	**<0.001**
Master’s degree or above	1.42 (0.80–2.53)	0.228	1.39 (0.83–2.33)	0.212	1.21 (0.79–1.84)	0.382	4.00 (2.11–7.57)	**<0.001**
**Health insurance**								
Medical insurance for urban employees and residents	Reference		Reference		Reference		Reference	
New rural cooperative medical insurance	1.38 (1.13–1.70)	**0.002**	1.55 (1.30–1.87)	**<0.001**	1.37 (1.18–1.59)	**<0.001**	1.21 (0.89–1.66)	0.221
Others	1.00 (0.81–1.24)	0.996	1.07 (0.88–1.30)	0.502	1.07 (0.90–1.26)	0.46	1.15 (0.82–1.63)	0.417
**Occupation**								
Service industry personnel	Reference		Reference		Reference		Reference	
Healthcare workers	2.54 (1.47–4.39)	**0.001**	1.95 (1.19–3.19)	**0.008**	2.08 (1.41–3.08)	**<0.001**	1.99 (1.27–3.12)	**0.003**
Civil servants and employees of enterprises/institutions	1.15 (0.72–1.83)	0.566	1.08 (0.69–1.69)	0.738	1.17 (0.81–1.70)	0.411	0.65 (0.36–1.18)	0.155
Pleasant	1.27 (0.86–1.87)	0.229	0.93 (0.65–1.34)	0.695	0.99 (0.74–1.33)	0.95	0.41 (0.26–0.66)	**<0.001**
Retired	1.10 (0.79–1.53)	0.575	1.00 (0.73–1.38)	0.991	0.97 (0.75–1.26)	0.808	0.42 (0.28–0.63)	**<0.001**
Unemployed/freelance	0.82 (0.55–1.24)	0.344	0.60 (0.41–0.87)	**0.008**	0.68 (0.49–0.93)	**0.016**	0.27 (0.14–0.55)	**<0.001**
Others	0.83 (0.57–1.21)	0.329	0.56 (0.39–0.80)	**0.001**	0.61 (0.46–0.82)	**0.001**	0.41 (0.24–0.71)	**0.001**
**Chronic disease**								
No	1.85 (1.54–2.23)	**<0.001**	1.51 (1.29–1.77)	**<0.001**	1.51 (1.33–1.71)	**<0.001**	1.24 (0.99–1.56)	0.063
Yes	Reference		Reference		Reference		Reference	

Statistically significant *p*-values were bold. Notably, the coverage rate of 4 doses only refers to the proportion of people who have received a total of four doses of COVID-19 vaccines in the whole population. aOR: adjusted odds ratio; CI: confidence interval.

## Data Availability

All data in the study are available from the corresponding author by request.

## Supplementary Materials

The following supporting information can be downloaded at: https://www.mdpi.com/article/10.3390/vaccines11040739/s1, Table S1: COVID-19 vaccination coverage rates stratified by dose among people aged 18–59 years old; Table S2: Demographic determinants associated with the COVID-19 vaccination coverage rates among people aged 18–59 years old; Table S3: Sensitivity analysis to explore demographic determinants associated with the COVID-19 vaccination coverage among all population.
