# Supplementary material for: COVID-19 Vaccination Coverage among 42,565 Adults Amid the Spread of Omicron Variant in Beijing, China
Source: Vaccines (Basel). 2023 Mar 27;11(4):739. doi: 10.3390/vaccines11040739 (PMC10146383; doi:10.3390/vaccines11040739)
Supplement: Supplementary file 1 [file vaccines-11-00739-s001.zip › vaccines-2288894-supplementary.pdf]

Table S1. COVID-19 vaccination coverage rates stratified by dose among people aged 18-59 years old.

Characteristics	≥1 dose			≥2 doses			≥3 doses			4 doses		
	**n (%)**	**95% CI**	**p value**	**n (%)**	**95% CI**	**p value**	**n (%)**	**95% CI**	**p value**	**n (%)**	**95% CI**	**p value**
**Total**	28,686 (95.5)	95.3-95.7		28,333 (94.3)	94.1-94.6		26,574 (88.5)	88.1-88.8		5050 (16.8)	16.4-17.2	
**Age (years)**			**<0.001**			**<0.001**			**<0.001**			**<0.001**
18-29	4029 (96.9)	96.3-97.4		3985 (95.8)	95.2-96.4		3627 (87.2)	86.2-88.2		732 (17.6)	16.5-18.8	
30-39	9103 (95.7)	95.3-96.1		8972 (94.3)	93.8-94.8		8304 (87.3)	86.6-87.9		1818 (19.1)	18.3-19.9	
40-49	7926 (96.4)	95.9-96.7		7839 (95.3)	94.8-95.7		7470 (90.8)	90.2-91.4		1510 (18.4)	17.5-19.2	
50-59	7628 (93.7)	93.2-94.3		7537 (92.6)	92.0-93.2		7173 (88.2)	87.4-88.8		990 (12.2)	11.5-12.9	
**Gender**			0.175			0.06			**0.003**			**<0.001**
Male	8987 (95.7)	95.3-96.1		8889 (94.7)	94.2-95.1		8381 (89.3)	88.7-89.9		1415 (15.1)	14.4-15.8	
Female	19,699 (95.4)	95.1-95.7		19,444 (94.2)	93.8-94.5		18,193 (88.1)	87.7-88.5		3635 (17.6)	17.1-18.1	
**Location**			0.101			**0.005**			**<0.001**			0.699
Urban	20,612 (95.4)	95.1-95.7		20,334 (94.1)	93.8-94.4		18,958 (87.7)	87.3-88.2		3622 (16.8)	16.3-17.3	
Rural	8074 (95.8)	95.4-96.2		7999 (94.9)	94.4-95.4		7616 (90.4)	89.7-91.0		1428 (16.9)	16.2-17.8	
**Education**			**<0.001**			**<0.001**			**<0.001**			**<0.001**

Junior high school and below	3694 (93.9)	93.1-94.6		3650 (92.8)	91.9-93.5		3458 (87.9)	86.8-88.9		296 (7.5)	6.7-8.4	
High school and technical secondary school	4896 (94.6)	93.9-95.2		4833 (93.4)	92.7-94.0		4556 (88.0)	87.1-88.9		588 (11.4)	10.5-12.2	
Bachelor degree	18,072 (96.2)	95.9-96.5		17,859 (95.1)	94.7-95.4		16,772 (89.3)	88.8-89.7		3914 (20.8)	20.3-21.4	
Master degree or above	2024 (94.7)	93.7-95.6		1991 (93.2)	92.0-94.2		1788 (83.7)	82.1-85.2		252 (11.8)	10.5-13.2	
**Health insurance**			**0.006**			**0.009**			**<0.001**			**<0.001**
Medical insurance for urban employees and residents	24,407 (95.7)	95.4-95.9		24,107 (94.5)	94.2-94.8		22,669 (88.8)	88.5-89.2		4669 (18.3)	17.8-18.8	
New rural cooperative medical insurance	2717 (94.9)	94.0-95.6		2688 (93.9)	92.9-94.7		2546 (88.9)	87.7-90.0		219 (7.6)	6.7-8.7	
Others	1562 (94.3)	93.1-95.3		1538 (92.8)	91.5-94.0		1359 (82.0)	80.1-83.8		162 (9.8)	8.4-11.3	
**Occupation**			**<0.001**			**<0.001**			**<0.001**			**<0.001**
Service Industry personnel	3082 (96.5)	95.8-97.1		3050 (95.5)	94.7-96.2		2860 (89.5)	88.4-90.6		337 (10.6)	9.5-11.7	
Health care workers	10,069 (97.6)	97.2-97.8		9957 (96.5)	96.1-96.8		9546 (92.5)	92.0-93.0		3002 (29.1)	28.2-30.0	
Civil servants and employees of enterprises/institutions	5070 (95.3)	94.7-95.8		5007 (94.1)	93.4-94.7		4662 (87.6)	86.7-88.5		739 (13.9)	13.0-14.8	
Pleasant	1736 (94.8)	93.7-95.8		1717 (93.8)	92.6-94.8		1645 (89.8)	88.4-91.2		146 (8.0)	6.8-9.3	
Retired	1683 (90.5)	89.1-91.8		1657 (89.1)	87.6-90.4		1529 (82.2)	80.4-83.9		82 (4.4)	3.5-5.4	

Unemployed/freelance	1667 (89.7)	88.2-91.0		1642 (88.3)	86.8-89.7		1462 (78.6)	76.7-80.5		81 (4.4)	3.5-5.4	
Others	5379 (95.2)	94.7-95.8		5303 (93.9)	93.2-94.5		4870 (86.2)	85.3-87.1		663 (11.7)	10.9-12.6	
**Chronic disease**			**<0.001**			**<0.001**			**<0.001**			**<0.001**
No	18,894 (96.7)	96.5-97.0		18,687 (95.7)	95.4-96.0		17,470 (89.5)	89.0-89.9		3471 (17.8)	17.2-18.3	
Yes	9792 (93.2)	92.7-93.7		9646 (91.8)	91.3-92.3		9104 (86.7)	86.0-87.3		1579 (15.0)	14.4-15.7	

Statistically significant p-values are bold. Notably, the coverage rate of 4 doses only refers to the proportion of people who have received a total of four doses of COVID-19 vaccines in the whole population.

CI: confidence interval

Table S2. **Demographic determinants associated with the COVID-19 vaccination coverage rates among people aged 18-59 years old.**

Characteristics	≥1 dose		≥2 doses		≥3 doses		4 doses	
	aOR (95% CI)	p value	aOR (95% CI)	p value	aOR (95% CI)	p value	aOR (95% CI)	p value
**Age (years)**								
18-29	0.96 (0.75-1.21)	0.705	0.91 (0.73-1.12)	0.354	0.55 (0.47-0.63)	**<0.001**	0.75 (0.66-0.84)	**<0.001**
30-39	0.79 (0.67-0.95)	**0.01**	0.75 (0.64-0.88)	**<0.001**	0.60 (0.53-0.67)	**<0.001**	0.92 (0.83-1.02)	0.106
40-49	1.23 (1.04-1.46)	**0.018**	1.16 (0.99-1.35)	0.059	1.02 (0.91-1.15)	0.743	1.06 (0.96-1.17)	0.232
50-59	Reference		Reference		Reference		Reference	
**Gender**								
Male	1.25 (1.10-1.41)	**0.001**	1.25 (1.12-1.40)	**<0.001**	1.26 (1.16-1.37)	**<0.001**	1.00 (0.93-1.08)	0.995
Female	Reference		Reference		Reference		Reference	
**Location**								
Urban	Reference		Reference		Reference		Reference	
Rural	1.33 (1.14-1.54)	**<0.001**	1.38 (1.21-1.58)	**<0.001**	1.45 (1.31-1.60)	**<0.001**	1.57 (1.46-1.70)	**<0.001**
**Education**								
Junior high school and below	Reference		Reference		Reference		Reference	
High school and technical secondary school	1.07 (0.88-1.29)	0.498	1.06 (0.89-1.27)	0.487	1.06 (0.93-1.22)	0.38	1.25 (1.07-1.47)	**0.005**
Bachelor degree	1.10 (0.90-1.34)	0.369	1.12 (0.93-1.34)	0.226	1.02 (0.88-1.17)	0.829	1.50 (1.28-1.76)	**<0.001**
Master degree or above	0.81 (0.61-1.08)	0.144	0.83 (0.65-1.08)	0.16	0.69 (0.57-0.83)	**<0.001**	0.89 (0.72-1.10)	0.288
**Health insurance**								

Medical insurance for urban employees and residents	Reference		Reference		Reference		Reference	
New rural cooperative medical insurance	1.18 (0.94-1.49)	0.163	1.15 (0.93-1.42)	0.198	1.04 (0.88-1.22)	0.661	0.67 (0.56-0.80)	**<0.001**
Others	0.89 (0.71-1.12)	0.326	0.88 (0.72-1.08)	0.229	0.71 (0.62-0.82)	**<0.001**	0.70 (0.59-0.83)	**<0.001**
**Occupation**								
Service Industry personnel	Reference		Reference		Reference		Reference	
Health care workers	1.57 (1.24-2.00)	**<0.001**	1.40 (1.14-1.73)	**0.002**	1.79 (1.55-2.07)	**<0.001**	3.33 (2.92-3.80)	**<0.001**
Civil servants and employees of enterprises/institutions	0.80 (0.63-1.01)	0.063	0.82 (0.66-1.01)	0.066	1.01 (0.87-1.17)	0.904	1.38 (1.20-1.60)	**<0.001**
Pleasant	0.61 (0.45-0.84)	**0.002**	0.63 (0.48-0.84)	**0.002**	0.77 (0.62-0.96)	**0.019**	0.81 (0.64-1.01)	0.063
Retired	0.50 (0.38-0.65)	**<0.001**	0.53 (0.41-0.68)	**<0.001**	0.57 (0.47-0.68)	**<0.001**	0.41 (0.31-0.53)	**<0.001**
Unemployed/freelance	0.31 (0.25-0.40)	**<0.001**	0.36 (0.29-0.44)	**<0.001**	0.43 (0.37-0.51)	**<0.001**	0.41 (0.32-0.52)	**<0.001**
Others	0.71 (0.57-0.90)	**0.004**	0.72 (0.58-0.88)	**0.001**	0.79 (0.68-0.90)	**0.001**	1.08 (0.94-1.25)	0.281
**Chronic disease**								
No	2.14 (1.89-2.43)	**<0.001**	2.03 (1.81-2.27)	**<0.001**	1.56 (1.44-1.70)	**<0.001**	1.05 (0.98-1.14)	0.182
Yes	Reference		Reference		Reference		Reference	

Statistically significant p-values are bold. Notably, the coverage rate of 4 doses only refers to the proportion of people who have received a total of four doses of COVID-19 vaccines in the whole population.

aOR: adjusted odds ratio; CI: confidence interval

Table S3. Sensitivity analysis to explore demographic determinants associated with the COVID-19 vaccination coverage among all population.

Characteristics	≥1 dose		≥2 doses		≥3 doses		4 doses	
	aOR (95% CI)	p value	aOR (95% CI)	p value	aOR (95% CI)	p value	aOR (95% CI)	p value
**Age (years)**								
	0.98 (0.97-0.98)	**<0.001**	0.97 (0.97-0.98)	**<0.001**	0.99 (0.98-0.99)	**<0.001**	1.00 (1.00-1.00)	0.414
**Gender**								
Male	1.19 (1.10-1.29)	**<0.001**	1.16 (1.08-1.25)	**<0.001**	1.14 (1.08-1.21)	**<0.001**	1.02 (0.95-1.09)	0.611
Female	Reference		Reference		Reference		Reference	
**Location**								
Urban	Reference		Reference		Reference		Reference	
Rural	1.39 (1.24-1.55)	**<0.001**	1.43 (1.30-1.58)	**<0.001**	1.40 (1.30-1.51)	**<0.001**	1.60 (1.48-1.72)	**<0.001**
**Education**								
Junior high school and below	Reference		Reference		Reference		Reference	
High school and technical secondary school	1.34 (1.20-1.49)	**<0.001**	1.46 (1.32-1.61)	**<0.001**	1.45 (1.34-1.57)	**<0.001**	1.48 (1.30-1.68)	**<0.001**
Bachelor degree	1.21 (1.07-1.36)	**0.002**	1.32 (1.18-1.47)	**<0.001**	1.26 (1.15-1.37)	**<0.001**	1.86 (1.62-2.14)	**<0.001**
Master degree or above	0.91 (0.73-1.14)	0.406	0.97 (0.79-1.18)	0.757	0.82 (0.71-0.95)	**0.009**	1.16 (0.96-1.41)	0.122
**Health insurance**								
Medical insurance for urban employees and residents	Reference		Reference		Reference		Reference	
New rural cooperative medical insurance	1.38 (1.18-1.60)	**<0.001**	1.42 (1.24-1.63)	**<0.001**	1.17 (1.05-1.30)	**0.004**	0.70 (0.60-0.81)	**<0.001**
Others	0.97 (0.83-1.13)	0.679	1.00 (0.87-1.15)	0.992	0.84 (0.76-0.93)	**0.001**	0.72 (0.62-0.84)	**<0.001**
**Occupation**								

Service Industry personnel	Reference		Reference		Reference		Reference	
Health care workers	1.59 (1.29-1.96)	**<0.001**	1.32 (1.09-1.59)	**0.004**	1.57 (1.37-1.79)	**<0.001**	3.02 (2.67-3.42)	**<0.001**
Civil servants and employees of enterprises/institutions	0.85 (0.69-1.04)	0.12	0.83 (0.69-1.00)	0.052	0.97 (0.85-1.11)	0.68	1.26 (1.10-1.45)	**0.001**
Pleasant	0.81 (0.65-1.02)	0.074	0.75 (0.61-0.93)	**0.007**	0.81 (0.70-0.95)	**0.008**	0.70 (0.57-0.85)	**<0.001**
Retired	0.73 (0.60-0.88)	**0.001**	0.77 (0.65-0.91)	**0.002**	0.70 (0.62-0.79)	**<0.001**	0.35 (0.29-0.41)	**<0.001**
Unemployed/freelance	0.40 (0.33-0.50)	**<0.001**	0.40 (0.33-0.49)	**<0.001**	0.46 (0.40-0.53)	**<0.001**	0.39 (0.30-0.49)	**<0.001**
Others	0.69 (0.57-0.83)	**<0.001**	0.61 (0.52-0.73)	**<0.001**	0.67 (0.59-0.76)	**<0.001**	0.97 (0.85-1.11)	0.663
**Chronic disease**								
No	1.73 (1.56-1.91)	**<0.001**	1.55 (1.42-1.69)	**<0.001**	1.32 (1.24-1.41)	**<0.001**	1.05 (0.98-1.13)	0.188
Yes	Reference		Reference		Reference		Reference	

Statistically significant p-values are bold. Notably, the coverage rate of 4 doses only refers to the proportion of people who have received a total of four doses of COVID-19 vaccines in the whole population.

aOR: adjusted odds ratio; CI: confidence interval
